# A review of existing scientific literature on welfare assessment of farmed species applied in commercial practice: identification of strengths, weaknesses, and areas for further development

**DOI:** 10.3389/fvets.2025.1589462

**Published:** 2025-06-19

**Authors:** Ingrid C. de Jong, Wijbrand Ouweltjes, Pol Llonch, Gerard E. Martin Valls, Heng-Lun Ko, Hans Spoolder, Ana C. Strappini

**Affiliations:** ^1^Wageningen Livestock Research, Wageningen University and Research, Wageningen, Netherlands; ^2^Department of Animal and Food Science, Universitat Autònoma de Barcelona, Cerdanyola del Vallès, Spain; ^3^Department of Animal Medicine and Surgery, University of Murcia, Murcia, Spain

**Keywords:** welfare assessment, protocol, indicators, livestock farming, welfare consequences

## Abstract

In the last decades, significant progress in welfare assessment of commercially farmed species has been achieved. Since then, various initiatives applied and improved existing protocols, or developed new ones for species like farmed rabbits or fish. This has resulted in a wide range of protocols, indicators and measures potentially lacking standardization and harmonization. However, standardized protocols are crucial for generating quantitative and comparable welfare data. In this literature review we (i) provide the state-of-the-art regarding application of welfare assessment protocols under commercial conditions for farmed species, (ii) their representation of the five welfare domains, and (iii) which animal-based welfare indicators have been applied. Further, (iv) we evaluate the alignment of welfare indicators as applied in scientific publications with highly relevant welfare consequences as defined by European Food Safety Authority (EFSA) for dairy cattle, pigs, broilers, and laying hens. Based on this, we (v) identify strengths and weaknesses regarding the domains covered and use of animal-based indicators, and define areas for further development. Most scientific publications focused on dairy cattle, followed by broilers, pigs and sheep. No publications were found for aquatic invertebrates, insects, fish species other than salmonids, and quails, highlighting the need for welfare assessment protocols for these species. Dairy cattle, horses, and sheep accounted for the highest number of unique indicators. Protocols generally covered all five welfare domains, with health indicators dominating. Animal-based welfare indicators were most prevalent. Common indicators across species were extracted and can be a starting point for the development of assessment protocols for novel species. Highly relevant welfare consequences as defined by EFSA were addressed. In conclusion, while welfare assessment protocols have been developed and tested under commercial conditions for many farmed species, assessment protocols for small-scale farmed species need attention. The wide variety of indicators extracted shows a lack of standardization and harmonization, risking divergence in indicators assessed between protocols. Attention should be given to define standardized welfare indicators per species, enabling comparable data collection related to important welfare issues and benchmarking to improve farm animal welfare.

## 1 Introduction

Welfare assessment of commercially farmed species is relevant to address societal concerns about farm animal welfare. These concerns are reflected in the results of the last European survey (1), where 84% of the surveyed citizens stated that the welfare of farmed animals should be better protected in their country than it is now. Information on the welfare status of farm animals can be used for food labeling to inform consumers (2), to inform farmers in order to improve the welfare of their animals, for benchmarking on animal welfare within production chains, and to support legislators. This calls for standardized animal welfare assessment protocols delivering harmonized and quantitative animal welfare data (3). Recent work conducted for the European Food Safety Authority (EFSA) (3) however indicates that there is currently a lack of quantitative, harmonized and validated data on welfare of farmed animals.

A welfare assessment protocol should contain comprehensive, valid and reliable indicators, and should be affordable and feasible to carry out in commercial practice (4). A welfare indicator can be defined as a measure or set of measures of a specific trait or state, which is specific to species and measurement context (5). In the last decades, considerable progress has been made in the development of welfare assessment protocols for farmed animals, particularly through European initiatives such as Welfare Quality (4) (dairy cattle, veal calves, pigs, broiler chickens, laying hens) and Animal Welfare Indicators (AWIN) for small ruminants, Equidae, turkeys and broiler chickens (6). Both Welfare Quality and AWIN addressed the multidimensional concept of animal welfare, requiring that all welfare domains are incorporated in the assessment protocols. Welfare Quality defined four welfare principles based on the five freedoms (i.e., good feeding, good housing, good health and appropriate behavior) and 12 underlying criteria, which should be fulfilled to meet the requirements regarding welfare (4) and the same framework was used by AWIN (6). Researchers applied these protocols in commercial conditions to collect information on the welfare of farmed species, to benchmark systems, and to further improve and refine existing welfare protocols. Following these developments, protocols have been developed that were for example aimed at being more feasible to perform under commercial conditions or considering the characteristics of the system (i.e., extensive or intensively housed) or covered species that were not addressed earlier [e.g., for rabbits (7)]. The Five Domains Model (8, 9) can also be used to provide a comprehensive framework for defining animal welfare (10). The first four domains (nutrition, environment, health, and behavior) focus on positive or negative subjective experiences of the animal, which contribute to the mental state of the animal, as evaluated in the fifth domain. Recently, the European Food Safety Authority (EFSA) panel on Animal Health and Welfare has investigated all potential welfare consequences that farmed animals can experience on farm and during transport (11). Moreover, based on their severity, duration and impact on the animals’ overall welfare they have defined “highly relevant” welfare consequences for some farmed species and also provided suggestions for indicators to monitor potentially impaired welfare (12–15). EFSA applies the “highly relevant welfare consequences” to their risk assessment models, which aim to guide public policies. These indicators should be animal-based, i.e., measure the response of an animal or an effect on an animal (16). Welfare Quality and AWIN protocols apply animal-based indicators where available or feasible but also include resource- or management-based welfare indicators (6).

For the main farmed species research efforts have resulted in a wide range of assessment protocols that are applied, but it is yet unclear whether these sufficiently cover all welfare domains, are properly standardized and to what extent these address the most important welfare issues. In addition, for species farmed on a smaller scale, welfare assessment protocols suitable for commercial conditions and covering all welfare domains are still lacking or are in an early stage of development. Moreover, it is unknown to what extent indicators in existing protocols can be linked to the (highly relevant) welfare consequences defined by EFSA. Therefore, the aim of this review paper is to provide the state-of-the-art knowledge with respect to (i) the application of welfare assessment under commercial conditions, (ii) the extent to which these are representing the five domains of welfare, and (iii) the proportion of animal-based over resource-based welfare indicators that are applied. In addition, for the four main farmed species, i.e., dairy cattle (12), pigs (13), laying hens (14) and broiler chickens (15), we aim to (iv) identify whether applied indicators cover highly relevant welfare consequences as defined by EFSA. Finally, we (v) aim to identify strengths and weaknesses based on the previous criteria (domains covered and animal-based over resource-based indicators) of existing welfare assessment protocols, and define areas for further development. Under (i) we included a wide range of farmed species and categories within them, i.e., dairy and beef cattle (including veal calves and dairy calves), rabbits, farmed fish (salmon, trout, carp, seabream, tuna), horses, small ruminants (i.e., sheep and goats), pigs, broiler chickens (including day-old chicks and broiler breeders), laying hens (including chicks, pullets, and laying hen breeders), turkeys, ducks, geese, quails, aquatic invertebrates (i.e., white leg shrimp, giant tiger prawn, common octopus), and insects (i.e., mealworms, crickets, and honey bees). The present review follows previous work published by Paulović et al. (3). Here we provide a more in-depth investigation of the results and discussion, and performed additional analyses of the data collected for the work of Paulović et al. (3).

## 2 Methods

### 2.1 Literature search strategy

Datasets were used generated for the purpose of another project, for which the search strategy is described in detail in Paulovic et al. (3). We used part of those earlier generated data [‘step 1 data’ (3)] and performed additional analyses as described below. The search strategy is briefly summarized here.

The literature review comprised a 10-year time frame, from 1-1-2013 until 1-1-2023, and aimed to find scientific papers reporting applications of welfare assessment protocols in animals under commercial conditions (i.e., excluding papers applying assessment protocols in experimental facilities). Seventeen categories of farmed species were included (Table 1). Regarding fish, aquatic invertebrates, and insects, two experts in the field were consulted to select the most farmed species. For the different species, different production stages were included (e.g., sows with piglets, gilts, broiler and layer breeders, day-old chicks and pullets and adult birds, rabbit does with kits and meat rabbits, etc.), further identified as “category within species.”

**Table 1:** Species included in the literature review.

Species	Limitations (if applicable)
Aquatic invertebrates	Only including octopus (*Octopus vulgaris*), white leg shrimp (*Penaeus vannamei*), and tiger prawn (*Penaeus monodon*)
Beef cattle and veal calves	
Broiler chickens	
Dairy cattle	
Dairy calves	
Ducks	
Fish	Only including salmon (*Salmo salar*), Rainbow trout (*Oncorhynchus mykiss*), common carp (*Cyprinus carpio*), Gilt-head seabream (*Sparus aurata*), European seabass (*Dicentrarchus labrax*), and Atlantic bluefin tuna (*Thunnus thynnus*). Search limited to the dominant production systems, i.e., husbandry of brood stock, rearing from larvae to fingerling and on growing to market size fish.
Geese	
Goats	
Horses	Horses for meat production, but as these can be sports and leisure horses, these categories were included. Welfare assessment during sports or races were excluded.
Insects	Only including meal worm, crickets (*Acheta domesticus* and *Gryllus bimaculatus*), and honey bees (*Apis mellifera*)
Laying hens	
Pigs	
Rabbits	
Sheep	
Turkeys	
Quails	

A Boolean literature review was performed using four databases: Web of Science (all fields), Scopus, CAB Abstracts, and Pubmed (restricted to title, key words and abstract). Searches were limited to English. For each species, specific search terms were applied, and these were combined with search terms related to welfare assessment under commercial conditions as described in Annex A of Paulović et al. (3). As an example, for dairy cattle, search terms related to the species (dairy cow, dairy cattle, milk* cow) were combined with terms related to commercial housing (farm, farmed, commercial farm, on-farm, herd, house, stable, slaughter, slaughter plant, abattoir, housing system, etc.). For all species these terms were combined with key-words related to welfare assessment: welfare assessment, welfare assessment method, welfare assessment framework; risk assessment, risk assessment method, risk assessment framework (all combined with welfare); welfare monitoring, welfare protocol, welfare assessment protocol, welfare indicator, welfare hazard, welfare label*, welfare scheme, welfare standard, welfare model, welfare guideline, welfare database, welfare data warehouse, welfare meta data, welfare initiative, welfare risk. For the number of initially found papers and the number of duplicates removed we refer to Appendix F in Paulović et al. (3). We used cross referencing and recently published EFSA scientific opinions (12–15) to identify additional scientific papers. Thereafter, duplicates were removed and records were screened for eligibility by reading the title and abstract. Eligible papers were studies performed under commercial conditions (commercial farms, slaughterhouses or both) and assessing the welfare of the species included in the study. Due to our search strategy, papers applying a selection of indicators and not the full welfare assessment protocol, and papers having a different purpose than welfare assessment (only), were also included.

### 2.2 Data extraction of welfare indicators for all farmed species

From each eligible paper, welfare indicators were extracted and included in Excel databases (one per species), each welfare indicator on a separate row. This means that welfare indicators that were used in more than one paper were extracted multiple times and thus represented in multiple rows in a species databases. We used the term ‘entry’ for each row in an Excel database. Welfare indicator names as used by the authors were included in the database, which could lead to spelling differences or different names used for indicators. Two indicators were considered different if they had different names even though they represented the same welfare consequence (the effect on the animal), or applied a similar scoring method. Information on the source was extracted and a number of attributes was linked to the entry as shown in Table 2. The full list of extracted attributes can be found in Appendix D of Paulović et al. (3). All extracted indicators were assigned to a welfare domain (9). For this we used the information in the respective papers, where indicators were often linked to a welfare domain (9) or to one of the four Welfare Quality principles (4). To the best of our knowledge, indicators within the Welfare Quality principle ‘appropriate behavior’ (4) were assigned to either the behavior or mental state domain (9). If authors did not provide clear links of indicators to a certain welfare domain we assigned these to a domain to the best of our knowledge. Per species/category, the various indicators for each welfare domain were assigned to four types: animal-based, environment-based, resource-based and management-based indicators. Animal-based indicators defined as a response of an animal or an effect on an animal. It can be taken directly from the animal or indirectly and includes animal records (16). Resource-based indicator defined as an evaluation of a feature of the environment in which the animal is kept or to which it is exposed (16). Management-based indicator defined as any indicator, tool, evaluation or management process an animal unit manager or stockperson applies (16). Environment-based indicator defined as any indicator describing the environmental conditions such as noise, dust, temperature, ventilation, light conditions, humidity and gas concentrations. Also the type of assessment protocol that was applied in a paper was extracted (e.g., Welfare Quality, AWIN).

**Table 2:** Description of attributes and information extracted from scientific papers.

Column name	Type of information	Description
Domain	Pre-defined options	Behavior, environment, health, mental state, or nutrition (8, 9)
Source	Free text	Author name(s) and year of publication
Doi or link	Free text	Doi or link to publication
Species	Pre-defined options (character)	Dairy cattle, dairy calves, beef cattle/veal calves, goats, sheep, pigs, chicken, turkey/duck/geese/quails, rabbits, horses, fish, aquatic invertebrates, insects
Category	Pre-defined options (character)	Category within a species, e.g., lactating, breeding, beef cattle, veal calves, sows, piglets, growing pigs, toms, broilers, laying hens, layer breeders, etc.
Indicator	Free text	The welfare indicator as has been named in the specific paper^1^
Type of indicator	Pre-defined options (character)	Animal-based, resource-based, management-based, or environment-based^2^
Assessment protocol name	Free text	If provided, the name of the assessment protocol applied, or a reference to the methodology, otherwise indicated as ‘own’
Manual or digital	Pre-defined options (characters)	Whether or not it is manually or digitally measured (digital including all types of sensors such as video, sound, etc)
Where measured	Pre-defined options (characters)	Is it measured on the farm or at slaughter, or both
Definition/criteria according to publication	Free text	Brief description of the definition of the indicator and/or the criteria used to score the indicator, according to the literature source
Other information	Free text	Free space to add other relevant information regarding the literature source, such as additions on the methods or definitions

^1^Welfare indicators extracted from the literature refer to both welfare consequences (the effect on the animal) and hazards (characteristics of the environment or management that indicate a welfare risk for the animal). Also, methods or measures were sometimes mentioned as indicators (e.g., qualitative behavior assessment, avoidance distance test) and papers may use the method name and sometimes the actual measures (e.g., number of animals withdrawing) of these methods. Information has been extracted as provided in the scientific paper. ^2^Animal-based: a response of an animal or an effect on an animal. It can be taken directly from the animal or indirectly and includes animal records (16); resource-based: an evaluation of a feature of the environment in which the animal is kept or to which it is exposed (16); management-based: any indicator, tool, evaluation or management process an animal unit manager or stockperson applies (16); environment-based: any indicator describing the environmental conditions such as noise, dust, temperature, ventilation, light conditions, humidity and gas concentrations.

### 2.3 Linking entries to welfare consequences for four main farmed species

Further analysis of the data collected for the work of Paulović et al. (3) was carried out for four main farmed animal categories, i.e., dairy cattle, pigs, broiler chickens, and laying hens. For those species, we assigned each entry as extracted from the scientific papers to one of the 33 welfare consequences as defined by EFSA for various farmed species (11), and differentiated between welfare consequences as defined to be highly relevant for the species according to the recent EFSA reports (12–15) (16, 16, 10 and 6 highly relevant welfare consequences for dairy cattle, pigs, laying hens and broiler chickens respectively) and other potential welfare consequences. Note that entries could have been linked to multiple welfare consequences. The definitions of EFSA (11) and the species-specific reports of EFSA (12–15) were used to relate entries to welfare consequences to the best of our knowledge. In addition to the 33 welfare consequences as defined by EFSA (9), we included two other categories. One ‘no specific welfare consequence’ where the entry could, to our opinion, not be linked to one or more of the 33 welfare consequences (9). The other category was ‘potential iceberg indicator’, with iceberg indicator defined as a key indicator providing an overall assessment of welfare, summarizing many measures of welfare and being easy to understand (17).

## 3 Results

### 3.1 All species

#### 3.1.1 Number of scientific papers, number of entries, and number of indicators per species/category

**Figure 1:**
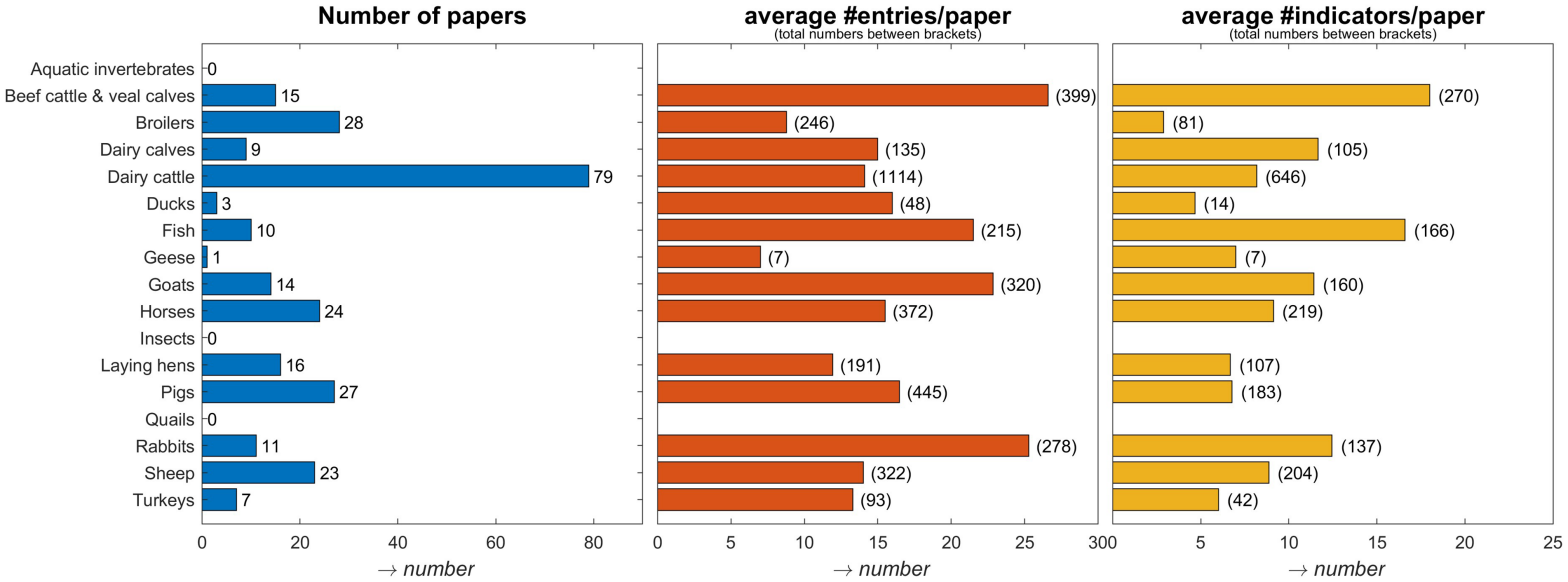
The number of papers that met eligibility criteria per species/category (left panel), the average number of entries per paper per species/category (middle panel), and the average number of unique indicators per species/category (right panel). In brackets after the bars in the middle panel the total number of entries for a species is shown, and in the right panel, in brackets the total number of unique indicators per species is shown. For fish, in the extracted papers, only Atlantic salmon/*Salmo salar*, European sea bass/*Dicentrarchus labrax*, Gilt-head seabream/*Sparus aurata* and Rainbow trout/*Oncorhynchus mykiss* were represented. No papers were extracted on broiler breeders and day-old chickens.

The literature search showed that most papers applying welfare assessment protocols under commercial conditions were found for dairy cattle (*n* = 79), followed by broilers (18), pigs (19) and sheep (20). Less than 10 papers were found for turkeys, ducks, and geese, and no papers for quails, aquatic invertebrates, and insects (Figure 1). Also, no papers were found for some categories within a species such as for broiler breeders and turkey breeders. For fish, the extracted papers represented four species (salmon, Rainbow trout, Gilt-head seabream, and European seabass) with most papers for salmon and Rainbow trout (four papers each). The average number of entries per paper shows that for some categories/species a wide range of indicators was included in the welfare assessments, such as for beef cattle and veal calves, goats, and rabbits, with on average more than 20 entries (i.e., 20 indicators measured) per paper, while for broilers and geese this was limited to less than 10 entries on average (Figure 1). The number of indicators was high for dairy cattle, beef cattle and veal calves, rabbits, horses, and sheep, indicating the application of many different indicators in the various papers (Figure 1). It should be noted that slightly different names could have been used for indicators having similar underlying scoring methods. Dairy cattle, horses and sheep account for the highest number of indicators (*n* = 646, 219, and 207, respectively) indicating a more complex and detailed welfare evaluation approach in comparison with geese and ducks (7 and 14 indicators, respectively).

#### 3.1.2 Distribution of indicators over the five welfare domains, and indicator type per domain and per species/category

**Figure 2:**
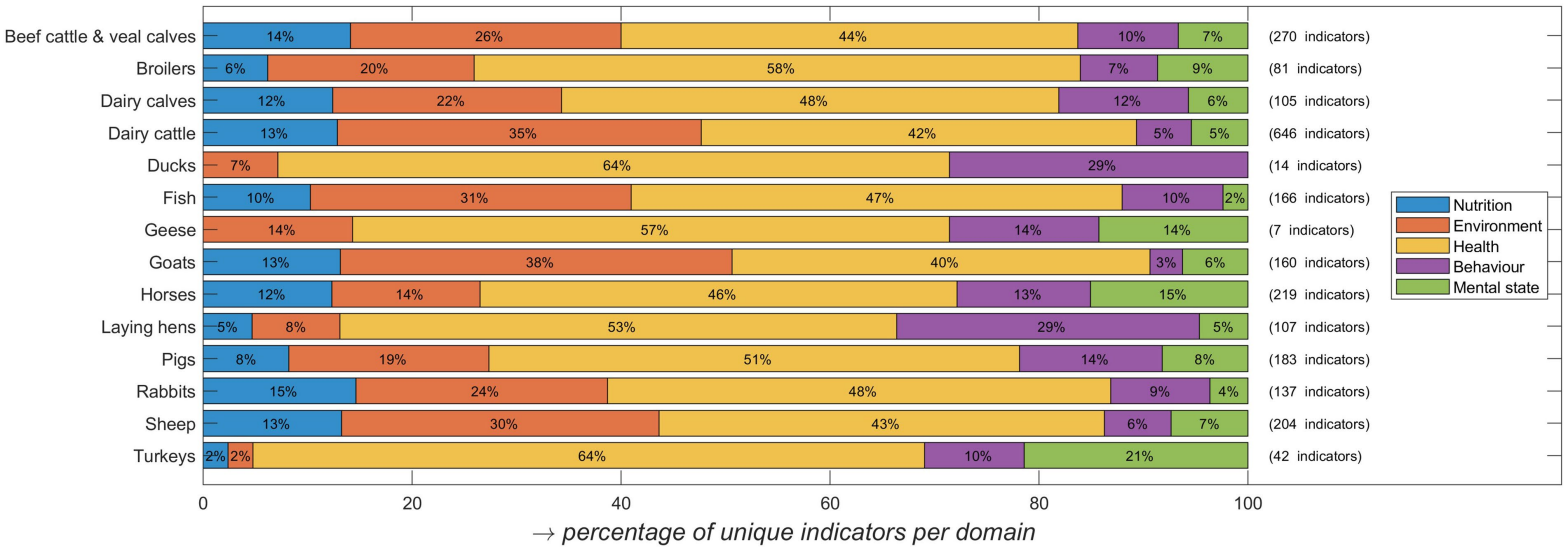
The percentage of unique indicators extracted for the five welfare domains: nutrition, environment, health, behavior, and mental state. The total number of indicators extracted per species/category is shown at the right of the bars in between brackets. Aquatic invertebrates, quails and insects are not included as no papers were extracted for these species.

For most species indicators were distributed across the Five Domains (9), with ducks and geese being an exception as for these categories the nutrition domain was not represented and for ducks also mental state was missing (Figure 2). Health-related indicators were dominating all protocols although for dairy cattle and goats many unique indicators were applied for the environmental domain. Fewer indicators associated with behavior and mental state were applied, and also for some species (dairy calves, pigs, broilers, laying hens) there were relatively few indicators representing the nutrition domain.

**Figure 3:**
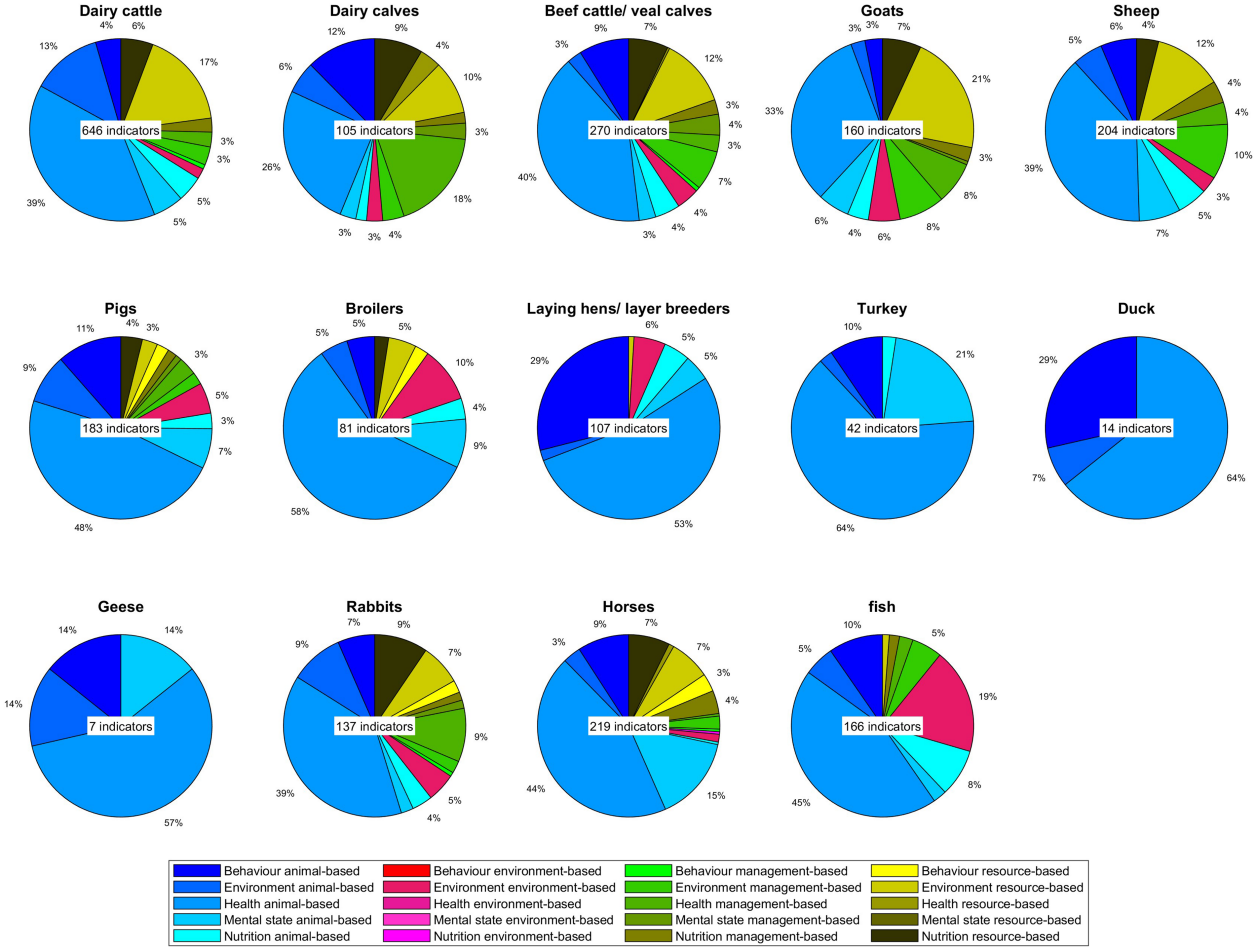
Overview of the type of indicator (animal-, environment-, resource-, management-based) per welfare domain for the different species/categories. Each pie chart shows the representation of indicator type per domain for a species/category, with each color within a species representing a different type of indicator (blue to cyan: animal-based; red to magenta: environment-based; green: management-based and yellow to black: resource-based). The dark–light variation within a color shows the presentation over the five domains. Aquatic invertebrates, quails and insects are not included as no papers were extracted for these species. Proportions lower than 3% are not indicated in figures.

Figure 3 provides an overview of welfare indicators categorized by type, and organized into the Five Domains of welfare (9). Each species or animal category is represented by a pie chart that shows the proportions of these categories of indicators. Across most species, animal-based indicators (blue) were most prevalent or even exclusively present (for turkeys, ducks, and geese). For ruminants, rabbits, and horses, relatively many resource-(black) and management-based (green) indicators were included. Fish and broilers had relatively many environment-based (magenta) indicators in the assessment protocols (Figure 3). Regarding the representation over the Five Domains (9), nutrition and environmental domains were often limited to resource-based indicators rather than animal-based indicators.

Table 3 presents an overview of the five most frequently extracted indicators per species and domain for the four main farmed species (dairy cattle, pigs, broilers and laying hens) and Supplementary Table 1 provides this for all other species. For several domains, similar indicators have been applied across species, such as the Qualitative Behavior Assessment (dairy cattle, dairy calves, sheep, pigs and broilers) and fear for humans (i.e., avoidance distance or flight distance) (all species except dairy calves, laying hens, ducks, and fish) for mental state domain, and body condition (scoring) (all species except dairy calves, laying hens, ducks, geese, and turkeys) for the nutrition domain. For the health domain, lameness or walking ability is within the top five most extracted indicators for several species (dairy cattle, beef cattle and veal calves, pigs, sheep, broiler chickens, turkeys and geese). Nasal and ocular discharge, cleanliness, diarrhea and fecal soiling are frequently applied for ruminants, horses, rabbits, and also in ducks. Differently, broilers, laying hens, and fish have more species-specific indicators in the health domain such as footpad dermatitis (broilers and laying hens) and fin condition in fish. In the behavior domain, social behavior (either negative or positive) was scored in several species (dairy cattle, dairy calves, sheep, pigs, turkeys, rabbits and horses) but generally, there was a large variation in indicators. E.g., in fish and dairy calves, several indicators appeared only once in the database. In laying hens, all indicators in the behavior domain related to feather damage, which was also assessed in ducks. Table 3 and Supplementary Table 1 confirm that resource-, management-, and environment-based indicators were particularly present in the environment and nutrition domains. For example, bedding (type, quality), dust, stocking density and cage sizes are used in the environment domain, and feeder space, water availability, water cleanliness, and type of feed in the nutrition domain.

**Table 3:** Overview of the top five indicators per domain as extracted from the scientific literature for dairy cattle, pigs, broilers and laying hens, and the number of times it was extracted from the various papers (displayed in brackets after the indicator).

Species (total entries)	Domain	Top five indicators (frequency)
Dairy cattle (1114)	Behavior	Agonistic behavior (4), Expression of normal behavior (2), Social behavio(u)r (2)^1^
	Environment	Time needed to lie down (11)*, Flooring* (8), Cleanliness of animals (7), Cleanliness of flank/upper legs (6)^2^, Cleanliness of lower legs (6)^2^, Cleanliness of udder (6)^2^, *Litter material* (6)^2^
	Health	Lameness (39), Nasal discharge (22), Ocular discharge (21), Diarrh(o)ea (17), Vulvar discharge (15)
	Mental state	Qualitative behavior assessment (8), Flight from humans test (6), Avoidance distance (5), Positive emotional state (3), Avoidance distance at the feeding rack (2)^2^, Flight distance (2)^2^
	Nutrition	Body condition score (18), Body condition (10), *Water provision* (6)^2^*, Cleanliness of water points* (6)^2^, *Cleanliness of water throughs* (6)^2^, *Functioning of water points* (6)^2^, *Size of drinking throughs* (6)^2^, State of nutrition (6)^2^
Pigs (445)	Behavior	Explorative behavior (7), Negative social behavior (5), Social behavior (5), Stereotypies (4), Positive social behavior (3)^2^, Use of enrichment material (3)^2^
	Environment	Bursitis (14), Panting (12), Manure on the body (11), Huddling (10), Shivering (9)
	Health	Lameness (17), Pumping (10), Rectal prolapse (10), Scouring (10), Skin condition (10)
	Mental state	Fear of humans (12), Qualitative behavior assessment (9), Time until the observer was surrounded by pigs with a radius of approx. 2 meters (2)^1^
	Nutrition	Body condition score (12)^1^
Broiler chickens (246)	Behavior	General behavior (2), *Outdoor access* (2)^1^
	Environment	Plumage cleanliness (14), *Litter Quality* (13), *Dust* (8)*, Stocking density* (7), Panting/huddling (6)
	Health	Footpad dermatitis (20), Walking ability (22), Hock burn (21), Mortality and culls (8), Mortality (7)
	Mental state	Qualitative behavior assessment (7), Avoidance distance test (6), Touch test (4)^1^
	Nutrition	*Drinker space* (7), Emaciation (5)^1^
Laying hens (191)	Behavior	Plumage back (12), Plumage tail (10), Plumage belly (7), Plumage breast (6), Plumage head (5)^2^, Plumage neck (5)^2^
	Environment	Dirty birds (3)^1^
	Health	Footpad lesions (6), keel bone fracture (6), Mortality (6), Keel bone deformation (5), Keel bone deviation (5)
	Mental state	Novel object test (2), Qualitative behavior assessment (2)
	Nutrition	3

Indicators displayed in italics are other than animal-based indicators (resource-, management-, or environment-based indicators). For all other species these data are shown in Supplementary Table 1.

^1^All other indicators were only applied once; ^2^These indicators had equal frequencies of extraction greater than 1 from the literature and are therefore all mentioned; ^3^Only indicators applied once.

#### 3.1.3 Welfare assessment protocols

Table 4 shows that for all species except rabbits and fish, either the assessment protocols developed within the Welfare Quality or within the AWIN project were applied most (or protocols derived from these and (slightly) adjusted). For rabbits, the WelFair protocol was the most common applied protocol (7). Table 4 also shows that for some species a large percentage of the papers applied other protocols. E.g., for dairy cattle there were many other protocols available, and for laying hens five other protocols were applied (Core Organic, AssureWel, LayWel, Aviary Transect, NorWel).

**Table 4:** Most applied assessment protocols for the different (categories of) species.

(Category of) species	Protocol name	% Papers in which this protocol was applied	Total number of papers
Dairy cattle	Welfare Quality (including adapted)	45	79
Dairy calves	Welfare Quality	22	9
Beef cattle/veal calves	Welfare Quality (including adapted)	33	15
Goats	AWIN (including adapted)	71	14
Sheep	AWIN	30	23
Pigs	Welfare Quality (including adapted)	55	27
Broilers	Welfare Quality (including adapted)	82	28
Laying hens	Welfare Quality (including adapted)	25	18
Turkeys	AWIN	22	7
Ducks	Abdelfattah et al. (26)	75	3
Rabbits	WelFair	27	11
Horses	AWIN	25	24
Fish	SWIM1.0	30	10

Geese are not represented in the table as information could only be extracted from one paper. Quails, aquatic invertebrates and insects are not represented as there were no papers found.

#### 3.1.4 Location of assessment (farm or slaughter plant) and manual or digital assessment

In the vast majority of the papers, for all species/categories, most assessments were performed on- farm, with limited evaluations taking place in other contexts, such as slaughter plants. Broilers had the highest proportion of indicators assessed at slaughter (17%). On the other hand, for dairy calves, goats, sheep, ducks, geese, rabbits, and horses, there were no assessments at the slaughter plant at all (data not shown). The majority of assessments were performed manually and only in few occasions instruments (e.g., to measure gas concentrations), video algorithms or other types of sensors were applied (data not shown).

### 3.2 Links between entries and welfare consequences for the four main animal categories

**Figure 4:**
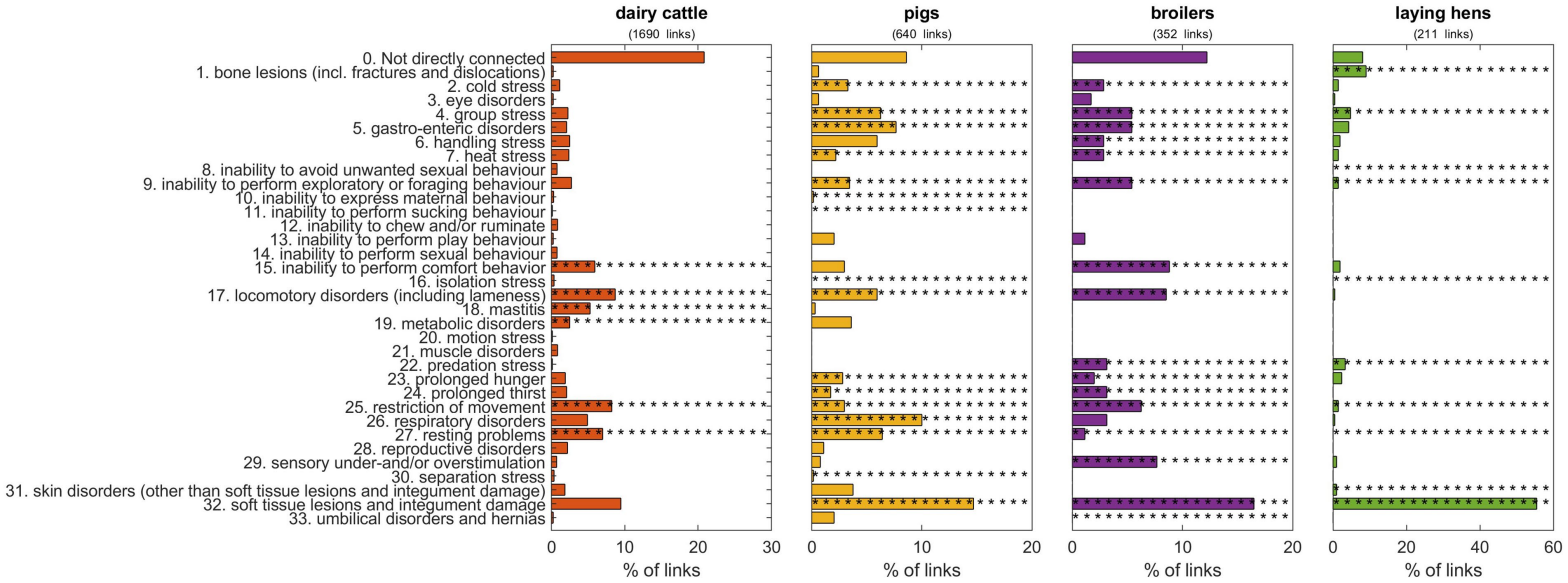
The number of entries per species, for the four main farmed species, in relation to the 33 welfare consequences as defined by EFSA (11) and the number of entries that could not be directly connected with one of these 33 welfare consequences (category 0, upper line). For each species, EFSA defined highly relevant welfare consequences (12–15) which are represented by asterisks.

Figure 4 shows the connection between the welfare consequences as defined by EFSA (11) and the entries as extracted from the papers for four major farmed species (broilers, laying hens, pigs, and dairy cattle). Note that entries can be linked to multiple welfare consequences. Results show species-specific considerations, as not all welfare consequences are applicable for each species (e.g., inability to express maternal behavior for broiler chickens) and not all welfare consequences are considered relevant to each category of species. For broiler chickens (352 links) and laying hens (211 links), the connection was largely on health and physical condition (e.g., integument damage for broiler chickens and laying hens, locomotory disorders for broilers, and fractures and dislocations for laying hens). For pigs, having 640 links in total, entries were more evenly distributed across multiple welfare consequences compared to both poultry species, and multiple categories were included in assessment protocols (piglets, sows, fattening pigs). Entries as extracted from pig papers were connected mainly with soft tissue and integument damage and respiratory disorders. On the other hand, in dairy cattle (1,690 links) health-related issues (e.g., mastitis) and locomotory disorders (e.g., lameness) were the most frequent entries linked to welfare consequences.

A high number of entries extracted from scientific literature were not linked to any defined welfare consequence as outlined by EFSA, particularly for broiler chickens and dairy cattle, and were defined as “not directly connected” (category 0). For broiler chickens and laying hens, this category mainly included mortality, culls and carcass rejections at the plant, which could not be directly connected to a particular welfare consequence. For dairy cattle and pigs, this category was more diverse, with entries related to health issues which were not further specified and many resource- or management-based indicators that could not be directly linked to welfare (examples are fence height, milking frequency and general behavior for dairy cattle). For dairy cattle, all highly relevant welfare consequences could be related to indicators present in the papers. For broiler chickens, umbilical disorders and hernias were not represented in the papers, but this is relevant to day-old chicks for which no protocols were found. For laying hens, resting problems, isolation stress and the ability to avoid unwanted sexual behavior were not included; the latter two being relevant to layer breeders. Although one paper included layer breeders, these welfare consequences were not addressed. Finally, for pigs, isolation stress and inability to perform sucking behavior were not represented by indicators included in papers, despite these are particularly relevant for specific categories of pigs.

Some relevant welfare consequences had links with just a few entries. Examples are separation stress and inability to perform maternal behaviors for pigs, skin disorders, eye disorders, locomotory disorders and sensory over- or under stimulation for laying hens, resting problems for broiler chickens and mastitis for dairy cattle.

## 4 Discussion

In the present paper we reviewed scientific literature over a 10-year period, reporting on the application of welfare assessment protocols in commercial conditions. The papers addressed a wide range of farmed species, and the gathered literature was used to identify strengths and weaknesses of existing assessment protocols, and to identify areas for further development.

The screening of scientific papers clearly showed a lack of information for novel farmed species such as insects and invertebrates, as no scientific papers on welfare assessment under commercial conditions could be found for these species. Insect farming, although increasingly applied, is still in its infancy and this also holds for studies on evidence of sentience and how to assess welfare in the various insect species (21, 22). Although there is evidence for sentience in decapods and cephalopods (23), there is a lack of welfare indicators for these species, and the development of welfare assessment protocols for aquatic invertebrates is still in an early stage (24, 25). Development and testing of protocols for these animals under commercial conditions is clearly an area for further exploration.

For species farmed on a relatively small scale (quail, common carp, Atlantic bluefin tuna) and for narrowly defined production categories within species (e.g., broiler breeders), no scientific papers on welfare assessment in commercial farms were found. Within the AWIN project, a welfare assessment protocol has been developed for turkeys (20), and more recently an assessment protocol has been developed for farmed rabbits based on the same principles as Welfare Quality (7), and initiatives were undertaken for farmed ducks and geese [e.g., Abdelfattah et al. (26) and Tremolada et al. (27)]. However, papers reporting application of welfare assessment under commercial conditions remain relatively scarce. For fish, results suggest that protocols for various farmed fish species are under development. The most important welfare risks and issues differ between farmed species, depending on the unique biology of the given species and the species-specific housing and management practices. However, when screening the top five indicators that have been applied across the various species, we observed common indicators in some domains (e.g., avoidance or flight distance to assess fear of humans in the mental state domain; lameness, injuries and fecal soiling in the health domain; body condition scoring in the nutrition domain). These common indicators can be used as a starting point for the development of assessment protocols for novel species. Regarding fish, although there are common aspects in farmed fish assessment protocols, different species have their own specific rearing conditions and welfare. Attempts have been made to design fish welfare protocols that can be applied across multiple fish farming systems (28), while other assessment protocols are specific for one single farmed fish species [e.g., salmon (19)].

The development of welfare assessment protocols initially focused on the major farmed species (dairy cattle, pigs, broilers and laying hens). Our results show that most scientific articles on the application of any welfare assessment protocol under commercial conditions refer to dairy cattle. A more detailed analysis of these protocols indicated a wide diversity of protocols applied in dairy cattle, where the application (or adaptation) of the Welfare Quality protocol stood out. This illustrates the relative great attention for welfare assessment of dairy cattle compared to the three other categories of main farmed species, i.e., pigs, broiler chickens and laying hens. For pigs, papers could include multiple species categories such as sows with piglets and weaned and fattening pigs, although the majority focused on fattening pigs. For dairy cattle, calves were included in a separate category. For horses and sheep, a comparable number of papers as compared to pigs, broilers, and laying hens was found. Although sport horses have been excluded from the study, the number of papers found for this species might have been biased as working horses as well as horses used for other purposes (i.e., recreational, draught power) were included, not necessarily being kept for purpose of meat production. However, horses originally kept for other purposes can end up in the food chain. Therefore, we selected papers on other categories than sport horses in our assessment. The number of horses assessed per farm was variable, but in general small. We did not specifically select papers on studies carried out in Europe, and for dairy and beef cattle nearly half of the studies was carried out in other countries (3). However, for the other species, nearly all the studies were performed in European countries, and the conclusions might therefore be more relevant to European conditions than in a global perspective because of the specific animal welfare legislation in force, or that not all the studies carried out outside Europe have been published in scientific journals.

The total number of indicators that were applied in the scientific papers, thus all indicators with a different name, was high, especially for dairy cattle, beef cattle and veal calves, sheep, and horses with more than 200 different indicators extracted for these species. Thus, many different indicators were applied in the various papers, although we also noticed that comparable indicators could have a slightly different name and were therefore extracted as being different (e.g., clean drinkers and cleanliness of water points). This suggests that there is a lack of standardization for the majority of species in the indicators that have been applied, which makes it difficult to compare outcomes of welfare assessments between studies. Further, on average, many entries per paper were found, meaning that per paper, many different indicators were scored. These numbers were especially high for beef cattle and veal calves, goats, rabbits, and fish, with over 20 indicators measured per paper on average. This suggests that multiple indicators could have been used in the assessment of the most relevant welfare consequences, or that many welfare consequences and/or risk factors were addressed in addition to the most relevant ones, especially in the health domain.

The drawback of welfare assessments including many indicators could be that it is time-consuming to complete the assessment, which may hamper application in practice due to high costs and lack of practical feasibility. This calls for the application of the so-called iceberg indicators. An iceberg indicator provides an overall assessment of welfare, somehow representing many measures of welfare (17). A well-functioning iceberg indicator is easy to implement and monitor (3). The inclusion of iceberg indicators in future welfare assessments could reduce the number of measures to be used, also reducing the time needed to carry out the assessment. These overall indicators can be used for indicative welfare assessment, enabling assessors to restrict conducting a more comprehensive assessment in case the threshold for iceberg indicators is exceeded and further inspection is needed. The literature reports examples of iceberg indicators for different species, such as stereotypies and frothy saliva in sows (18); absence of prolonged thirst in dairy cows (29); body condition, fleece cleanliness, color of the eye mucous membrane, flight distance and qualitative behavior assessment in sheep (30); mortality, feather cleanliness and walking ability in broiler chickens, and mortality, feather damage and keel bone damage in laying hens (31). Development of sensor technologies enables the development of standardized iceberg indicators that can be routinely collected, e.g., at slaughter, such as footpad lesions for broiler chickens (15). The development of sensor technologies for automated assessment of welfare indicators is very promising in general, as it may lead to substantial reduction in the time needed for assessment, enabling more continuous monitoring during the farm stage and early warning of emerging welfare problems. Few papers already included sensors to assess welfare under commercial conditions, such as optical flow patterns indicative of hock burn and mortality (32) and activity patterns indicative of hock burn and footpad dermatitis (33) in broiler chickens. A recent study that investigated the implementation of welfare assessments using sensor technologies found that a minority (one out of 19) of welfare schemes uses data from sensors, and this was limited to health data (34). This reveals the low degree of implementation of automatic measurements in routine welfare assessment.

Another explanation for the large number of unique indicators found for several species may be that protocols have been adapted due to experiences over time. Protocols resulting from projects such as Welfare Quality® and AWIN have been initiatives offering a scientifically grounded framework for assessing welfare in commercial farms, providing tools to benchmark and improve animal welfare across different systems and comparisons between farms. However, in many cases these protocols have been adapted to be feasible for commercial application, considering factors such as time constraints and farm resources or local farming conditions (i.e., in extensive farming systems). This was illustrated by the finding that not only the original protocols were applied, but also protocols derived from the original ones and customized to local conditions. Although this may lead to more feasible protocols or better indicators, it also reduces standardization and hampers comparison based on quantitative results. One example of this is the large variety in laying hen welfare assessment protocols in addition to Welfare Quality [e.g., Vasdal et al. (35) and Rorvang et al. (36)]. On the other hand, despite differences between laying hen protocols, a set of common indicators related to the most important welfare issues was included in the various laying hen studies, such as plumage damage scores, injury scores and keel bone fractures and deviations scores, indicating a starting point for standardization. There is clearly a need for a selection of a set of valid and feasible key welfare indicators for each species addressing the most important welfare consequences and all welfare domains. This selection should be based on consensus between stakeholders and should be re-evaluated on a regular basis to update with new developments in research.

For most species, all five welfare domains were represented with indicators, apart from ducks and geese where there were no indicators in the nutrition domain. For these species welfare assessment appears to be still in a premature stage of development, given the lack of papers on application and the fact that the papers found were from recent years. The relatively large number of indicators in the health domain for nearly all species can be explained by several reasons. First of all, the health domain has a broad definition, including injuries, disease, poor physical fitness and functional impairment (9). It includes pain induced by management procedures such as injuries according to Welfare Quality (4), and thus needs to be addressed by multiple indicators unless iceberg indicators are used. As welfare assessment protocols sometimes seem to be developed from a veterinarian view point, this may also explain the focus on disease, which seems to especially be the case in dairy cattle where a long list of unique health indicators was found. Further, injuries or diseases might be easier or more feasible to assess (and already included in standard veterinary protocols) than indicators in the other domains. This seems especially the case for indicators within the mental state and behavior domain. Behavioral observations are time consuming and there seems to be a lack of easy applicable indicators in the domain addressing the most important welfare consequences, reflected in less standardization in indicators between studies. E.g., in dairy calves or fish, few and different indicators between studies were found, clearly indicating an area for further development. Automated scoring of behavior using sensor technology may help to better address the behavior in welfare assessment protocols. For mental state, apart from the Qualitative Behavior Assessment which is applied in several species [e.g., Czycholl et al. (37), Gutmann et al. (38), and Phythian et al. (39)], assessment of emotions is still under development, in particular regarding positive emotions (40). Although research regarding assessment of positive emotions is in progress [e.g., Krugmann et al. (41), Laurijs et al. (42), and Papageorgiou et al. (43)] there is a lack of feasible and valid indicators to be applied in practice. A desk study within the same project as the current literature review showed several research initiatives in this area but also identified that more efforts to develop feasible indicators for positive welfare are needed (3).

The representation of indicators across the Five Domains reveals a limitation in the nutrition and environmental domains. These domains often rely on resource-based indicators (e.g., feed availability, water quality) rather than animal-based indicators (e.g., body condition, hydration status), restricting the depth of welfare evaluation in these areas. Despite resource-based indicators providing critical baseline information (e.g., availability of feed), they may not fully reflect the animal’s response (e.g., weight gain). Similarly, environment-based indicators, like ventilation (e.g., NH_3_ concentration) and space allowance (e.g., stocking density) are prioritized over animal-based indicators like signs of heat stress (e.g., panting). The reliance on resource- and environment-based indicators in certain domains can limit the accuracy and relevance of the welfare evaluation. Animal-based indicators are crucial for understanding the welfare state of an animal or group of animals. Results also showed a relatively high representation of management-based and environment-based indicators for all categories of ruminants in comparison to other species, sometimes in addition to animal-based indicators. Examples are registration of pasture access, cubicle size, pasture management, and management around weaning. This could be helpful to improve housing and management in relation to welfare.

Linking the indicators as represented in the literature to the full list of welfare consequences as defined by EFSA (11), and to the welfare consequences identified as ‘highly relevant’ for dairy cattle, pigs, broilers and laying hens (12–15), showed that most of the highly relevant welfare consequences per species could be linked to indicators as present in the screened studies. However, some widely used indicators could not be linked to highly relevant welfare consequences, especially for dairy cattle (e.g., general health problems, health indicators in blood, culled cows, clinical examination scores). The reason could be that that EFSA’s selection of highly relevant welfare consequences is based on their severity, duration, and frequency, and for example, severe but not frequently occurring welfare consequences were included in the papers. Nevertheless, a thorough screening of the indicators and possibly a selection of those linked to the most relevant welfare consequences could help to increase the feasibility of the protocols for commercial practice. Further, for each of the four main species, indicators were included that could not be linked to a specific welfare consequence but that are obvious indicators of impaired welfare such as condemnations, culls, and mortality. These can possibly be used as iceberg indicators leading to a more in-dept assessment when certain thresholds are exceeded.

## 5 Conclusion

A review of the scientific literature from 2013 to 2023 showed that for several farmed species, welfare assessment protocols have been developed and welfare assessments, either using a full assessment protocol or a selection of indicators, were applied on commercial farms. Although Welfare Quality and AWIN protocols were most often applied, there are many other welfare assessment methods used in scientific studies. The wide range of indicators applied, especially under the health domain, hampers comparison of quantitative data and calls for harmonization in data collection. The multidimensional aspect of animal welfare has been addressed for each species, although the behavior and mental state domains require effort in development of valid animal-based indicators that can be applied in practice. Similarly, the nutrition and environment domains often lack animal-based indicators and require further development. New protocols for less commonly farmed species requires further attention, such as poultry species including ducks, geese, quails, and certain sub-categories such as broiler breeders or day-old chicks, fish (particularly species that were not present in the literature), aquatic invertebrates, and insects. The present review shows that welfare assessments are diverse. In future studies, attention should be given to define and apply a standardized set of welfare indicators per species. The latter is needed to enable comparison of data across countries and initiatives, which is the basis of improvement of farm animal welfare by, e.g., enabling benchmarking of farms and production systems.
