# Supplementary material for: A review of existing scientific literature on welfare assessment of farmed species applied in commercial practice: identification of strengths, weaknesses, and areas for further development
Source: Front Vet Sci. 2025 Jun 19;12:1589462. doi: 10.3389/fvets.2025.1589462 (PMC12225303; doi:10.3389/fvets.2025.1589462)
Supplement: Supplementary file 1 [file Table_1.docx]

**Supplementary Table 1. Overview of the top 5 of indicators per domain as extracted from the scientific literature for dairy calves, beef cattle and veal calves, sheep, goats, turkeys, ducks, geese, rabbits, horses and fish, and the number of times it was extracted from the various papers (displayed in brackets after the indicator). For dairy cattle, pigs, broiler chickens and laying hens this information is shown in Table 2 in the paper.**

| Species | Domain | Indicator-top 5 (frequency) |
| --- | --- | --- |
| Dairy calves | Behaviour | ^1^ |
|  | Environment | Ability to turn around (3), *Calving area (3)*, ^3^ |
|  | Health | *Time of first colostrum (4), Age at dehorning (3)^2^, Amount of colostrum (3)^2^, Disinfection of calf navels (3)^2^, Method used to dehorn (3)^2^, Use of pain relief for dehorning (3)^2^* |
|  | Mental state | Qualitative behaviour assessment (3), ^3^ |
|  | Nutrition | *Weaning age (6), Adequate amount of milk (3), Frequency of feeding milk (3),* ^3^ |
| Beef cattle and veal calves | Behaviour | Falling (3), Running (3), Stumbling (3), Agonistic behaviour (2), Vocalisation (2) |
|  | Environment | Dirty body (5), Cleanliness (5), *Absence of hazardous objects/environment (3)^2^, Access to pasture (3)^2^, Dogs noise around the yard (3)^2^, noise of equipment/machinery (3)^2^, Noise of handlers (3)^2^, shade (3)^2^, Yarding frequency (3)^2^* |
|  | Health | Nasal discharge (10), Ocular discharge (9), Lameness (8), Diarrhoea (7), Mortality (7) |
|  | Mental state | Avoidance distance (4), fearful/agitated(3), *hitting (3), mis-catching (in chute/race) (3), tail twisting (3)* |
|  | Nutrition | Body condition score (11), *Distance and availability of water (3)*, Rumen fill (3), Bloated rumen (2)^2^, *Cleanliness of water points (2)^2^, distance to grazing (2)^2^* |

Table continued on the next page

| Species | Domain | Indicator-top 5 (frequency) |
| --- | --- | --- |
| Goats | Behaviour | Oblivion (10), ^3^ |
|  | Environment | Thermal stress (5), panting score (3), shivering score (3), *access to pasture (2)^2^, frequency of bedding replacement (2)^2^, illumination (2)^2^, ventilation (2)^2^* |
|  | Health | Nasal discharge (11), Ocular discharge (11), abscesses (10), faecal soiling (10), hair coat condition (10) |
|  | Mental state | Latency to first contact test (5), Qualitative behaviour assessment (5), Avoidance distance test (2), familiar human approach (2), handling test (2) |
|  | Nutrition | Body condition score (12), Kneeling at the feeding rack (7), Queuing at drinking (6), Queuing at feeding (6), *Type of drinkers (2)^2^, Water availability (2)^2^* |
| Sheep | Behaviour | Social withdrawal (6), Stereotypies (5), Abnormal behaviour (2) Demeanour (2), ^3^ |
|  | Environment | Fleece cleanliness (9), Panting (6), *Access to shade and shelter (4), Stocking density (3)*, Fleece condition (2), *Indoor space allowance (2),* Shivering (2) |
|  | Health | Lameness (15), Tail length (5), Hoof overgrowth (5), excessive itching (4)^2^, Faecal soiling (4)^2^, Fleece quality (4)^2^, Mucosa colour (4)^2^, Skin lesions (4)^2^ |
|  | Mental state | Qualitative behaviour assessment (9), Familiar human approach (3), Flight distance (2), Human approach test (2), ^3^ |
|  | Nutrition | Body condition score (7), *Water availability (5)*, Body condition (2), ^3^ |
| Turkeys | Behaviour | Featherless (5), Aggression towards mate (4), mounting (3), mating (1) |
|  | Environment | Dirty (5) |
|  | Health | Terminally ill (5)^2^, Small (5)^2^, Sick (5)^2^, Lame (5)^2^, Immobile (5)^2^, dead (5)^2^ |
|  | Mental state | Human interaction (3) |
|  | Nutrition | Average carcass weight (1) |
| Ducks | Behaviour | Feather quality (9), Feather quality and cleanliness (6), Inversion damage (1), Inversion rubbing (1) |
|  | Environment | Feather cleanliness (6) |
|  | Health | Blood on feather (6), Eye condition (5), Nostril condition (5), Dead (4)^2^, Gait (4)^2^, Lethargic (4)^2^ |
|  | Mental state | ^4^ |
|  | Nutrition | ^4^ |

Table continued on the next page

| Species | Domain | Indicator-top 5 (frequency) |
| --- | --- | --- |
| Geese | Behaviour | Feather pecking (1) |
|  | Environment | Plumage dirtiness (1) |
|  | Health | Broken wings (1), Feather irregularities (1), Immobility (1), Twisted wings (1) |
|  | Mental state | Handling test (1) |
|  | Nutrition | ^4^ |
| Rabbits | Behaviour | Abnormal behaviours (4), Negative social behaviour (4), Activity (2)^2^, *Enrichment material (2)*^2^, Interaction: sneeze or groom each other (2)^2^, Isolated animals *(2)*^2^, *Nesting material (does 24 h prior to parturition) (2)*^2^, stereotyping (2)^2^ |
|  | Environment | Dirty animals (6), *Stocking density (5)*, *Dust (4), Height of the cage (4),* Wet animals (4) |
|  | Health | Mortality (7), Diarrhea (6), Coughing (4)^2^, Culling (4)^2^, Fallen ears (4)^2^, Gait score (4)^2^, Mange (4)^2^, Nasal discharge (4)^2^, Neck torsions (4)^2^, Ocular discharge (4)^2^, pododermatitis (4)^2^, Sneezing (4)^2^, Wounds on the body (4)^2^, Wounds on the ears (4)^2^ |
|  | Mental state | Human approach test (2), Nervous (2), *Touching the kits (2), Training of personnel (2)* |
|  | Nutrition | Body condition (4), *Cleanliness of the drinkers (4), Cleanliness of the feeders (4)*, Body weight (3), *Functioning of the drinkers (3)* |
| Horses | Behaviour | Stereotypies (9), Social interaction (3), *Enrichment for feed-seeking behaviour (2),* Occurrence of unwanted behaviour (2), *Visual horizon in stalls (2)* |
|  | Environment | Bedding (3), Box dimensions (2)^2^, Collisions or slipping when moving from stable (2)^2^, *exercise (2)^2^*, Fecal soiling on the rump and ventral-lateral abdomen (2), *Mould in stable (2)^2^, Noise level (2)^2^*, *Rug cleanliness (2)^2^, Sum of relative humidity and T in stable (2)^2^,  Thermal comfort (2)^2^, Paddock surface quality (2)^2^* |
|  | Health | Lameness (10), Nasal discharge (8), Ocular discharge (8), Coughing (6)^2^, Hair coat condition (6)^2^, Signs of hoof neglect (6)^2^ |
|  | Mental state | Avoidance distance test (7), Forced human approach test (5), Qualitative behaviour assessment (5), Voluntary human approach test (4), Fear response (3) |
|  | Nutrition | Body condition score (15), *Water provision (4), Functioning of drinker (2)^2^, roughage fed without water (2)^2^, Time with available roughage (2)^2^, water cleanliness (2)^2^* |

# Table continued on the next page

| Species | Domain | Indicator-top 5 (frequency) |
| --- | --- | --- |
| Fish | Behaviour | ^1^ |
|  | Environment | *Stocking density (4), Water temperature (4),* *Disturbances (3), Lighting (2), Salinity (2), Water oxygen level (2)* |
|  | Health | Fin condition (4), Mortality (4), Cataract (3)^2^, Fin damage (3)^2^, Skin condition (3)^2^, Vertebral deformity (3)^2^ |
|  | Mental state | Blood cortisol (2), ^1^ |
|  | Nutrition | Emaciation (6), Condition factor (4), Feed conversion ratio (3), Appetite (2)^2^, Body weight (2)^2^, Feed intake (2)^2^, Specific growth rate (2)^2^, total length (2)^2^ |

Indicators displayed in italics are not animal-based indicators, but resource-, management- or environment-based indicators. ^1^ Only indicators applied once; ^2^ These indicators had equal frequencies of extraction greater than 1 from the literature and are therefore all mentioned; ^3^ All other indicators were only applied once; ^4^ No indicators extracted within this domain.
